# The role of isochrony in speech perception in noise

**DOI:** 10.1038/s41598-020-76594-1

**Published:** 2020-11-11

**Authors:** Vincent Aubanel, Jean-Luc Schwartz

**Affiliations:** CNRS, GIPSA-lab, University of Grenoble Alpes, Grenoble, France

**Keywords:** Language, Cortex

## Abstract

The role of isochrony in speech—the hypothetical division of speech units into equal duration intervals—has been the subject of a long-standing debate. Current approaches in neurosciences have brought new perspectives in that debate through the theoretical framework of predictive coding and cortical oscillations. Here we assess the comparative roles of naturalness and isochrony in the intelligibility of speech in noise for French and English, two languages representative of two well-established contrastive rhythm classes. We show that both top-down predictions associated with the natural timing of speech and to a lesser extent bottom-up predictions associated with isochrony at a syllabic timescale improve intelligibility. We found a similar pattern of results for both languages, suggesting that temporal characterisation of speech from different rhythm classes could be unified around a single core speech unit, with neurophysiologically defined duration and linguistically anchored temporal location. Taken together, our results suggest that isochrony does not seem to be a main dimension of speech processing, but may be a consequence of neurobiological processing constraints, manifesting in behavioural performance and ultimately explaining why isochronous stimuli occupy a particular status in speech and human perception in general.

## Introduction

A fundamental property of mammalian brain activity is its oscillatory nature, resulting in the alternation between excitable and inhibited states of neuronal assemblies^1^. The crucial characteristic of heightened excitability is that it provides, for sensory areas, increased sensitivity and shorter reaction times, ultimately leading to optimised behaviour. This idea formed the basis of the Active Sensing theoretical framework^2,3^ and has found widespread experimental support.

Oscillatory activity in relation to speech, a complex sensory signal, has initially been described as speech entrainment, or tracking, a view which proposes that cortical activity can be matched more or less directly to some characteristics of the speech signal such as the amplitude envelope^4,5^. The need to identify particular events to be the support of speech tracking has in turn prompted the question of which units would oscillatory activity entrain to. The syllable has usually been taken as the right candidate^6,7^, given the close match, under clear speech conditions, between the timing of syllable boundaries and that of the amplitude envelope’s larger variations. These conditions are however far from being representative of how speech is usually experienced: connected speech is notoriously characterised by the lack of acoustically salient syllable boundaries^8^.

In parallel, early works on speech rhythm, also inspired by evident similarities with the timing of music^9^, have led scholars to focus on periodic aspects of speech. The isochrony hypothesis extended impressionistic descriptions of speech sounding either morse-like or machine gun-like^10,11^, and led to the rhythmic class hypothesis^12^, stating that languages fall into distinct rhythmic categories depending of which unit is used to form the isochronous stream. Two main classes emerged, stress-timed languages (e.g., English) based on isochronous feet and syllable-timed languages (e.g., French) which assume equal-duration syllables. Still, the isochrony hypothesis and the related rhythmic class hypothesis, in spite (or by virtue) of their simple formulation and intuitive account, have been the source of a continuous debate (see review in^13^).

The way current theories are formulated as reviewed above, isochrony in speech would present some advantages: speech units delivered at an ideal isochronous pace would be maximally predictable and lead to maximum entrainment, through alleviating the need for potentially costly phase-reset mechanisms^14^. However, naturally produced speech is rarely isochronous, if at all, and this departure from a hypothetical isochronous form, that is, variation of sub-rhythmic unit durations is in fact used to encode essential information at all linguistic (pre-lexical, lexical, prosodic, pragmatic, discursive) and para-linguistic levels. Two apparently contradictory hypotheses are therefore at play here, the first one positing a beneficial role for isochrony in speech processing, and the second one seeing natural speech timing as a gold standard, with any departure from it impairing its recognition.

In this study we attempt to disentangle the role of the two temporal dimensions of isochrony and naturality in speech perception. We report on two experiments conducted separately on spoken sentences in French and English, each representative of the two rhythmic classes. For this aim, we exploited the Harvard corpus for English^15^ and its recently developed French counterpart, the Fharvard corpus^16^. Both corpora contain sentences composed of 5–7 keywords, recorded by one taker for each language. Sentences were annotated at two hierarchical rhythmic levels: the *accent group* level and the *syllable* level, respectively forming the basis of the two main language rhythmic classes mentioned above. We retimed naturally produced sentences to an isochronous form (or a matched anisochronous form, see hereafter) by locally compressing or elongating speech portions corresponding to rhythmic units at the two corresponding levels (accent group and syllable). Retiming was operationalised around P-centres, that is, the time at which listeners report the occurrence of the unit^17,18^, and which provide crucial pivotal events at the meeting point between bottom-up acoustic saliency cues and top-down information about the onset of linguistic units. Unmodified time onsets of either accent (acc) or syllable (syl) rhythmic units served as a reference for the natural rhythm (NAT) condition, from which isochronous (ISO) and anisochronous (ANI) conditions were defined. Altogether, this provided 5 temporal versions of each sentence in each corpus: the unmodified natural version (NAT), the isochronous stimuli at the accent (ISO.acc) and syllable (ISO.syl) levels, and the anisochronous stimuli at the accent (ANI.acc) and syllable (ANI.syl) levels. The ANI conditions were controls for the ISO conditions through the application of identical net temporal distortions from the NAT sentences though in a non-isochronous way (see “Methods”).

We then evaluated the consequences of these modifications of naturalness towards isochrony in the ability of listeners to process and understand the corresponding speech items. Sentence stimuli were mixed with stationary speech-shaped noise to shift comprehension to below-ceiling levels. Then, for both languages separately, the set of the five types of sentences in noise was presented to native listeners, and the proportion of recognised keywords was taken as the index of the intelligibility of the corresponding sentence in the corresponding condition. We show that naturalness is the main ingredient of intelligibility, while isochrony at the syllable level—but not at the accent group level, whatever the rhythmic class of the considered language—plays an additional though quantitatively smaller beneficial role. This provides for the first time an integrated coherent framework combining predictive cues related to bottom-up isochrony and top-down naturalness, describing speech intelligibility properties independently on the language rhythmic class.

## Results

### Natural timing leads to greater intelligibility than either isochronously or anisochronously retimed speech in both languages

**Figure 1:**
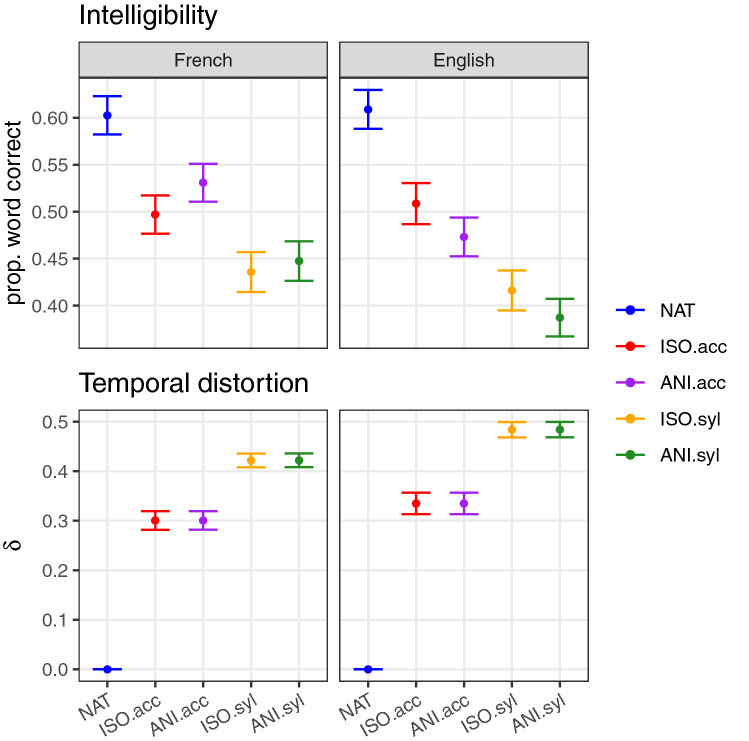
Top panel: proportion of words correctly recognised in each experimental condition for French and English. Error bars show 95% confidence intervals over 26 and 27 subjects in the two languages respectively. Bottom panel: average sentence temporal distortion ($\delta$ function computed on speech units matching the temporal condition, see Eq. (1)). By construction, temporal distortion is null for natural sentences (NAT condition) and identical for isochronously and anisochronously retimed sentences at a given rythmic level, that is, in ISO.acc and ANI.acc conditions on one hand, and in ISO.syl and ANI.syl conditions on the other hand. Error bars show 95% confidence intervals over 180 sentences for French and English. Data for English was previously reported in^19^.

We first report the effect of temporal distortion on intelligibility, separately by retiming condition, for the two languages. Figure 1 shows intelligibility results as the proportion of keywords correctly recognised by French and English listeners (top panel) and the temporal distortion applied to sentences in each condition (bottom panel, see “Methods”, Eq. (1) for computation details). Net temporal distortion from natural speech at the condition level appears to be reflected in listeners’ performance, with increased temporal distortion associated with decreased intelligibility in both languages.

Extending the analysis done for the English data and reported in^19^, we fitted a generalised linear mixed-effect model to the French data. Table 1 gathers the results of simultaneous generalised hypotheses on the condition effects formulated separately for each language.

**Table 1:** Simultaneous generalised hypotheses tests for the effect of condition on intelligibility, formulated on two independent models for French and English respectively. From left to right: comparison tested and, for each language, comparison estimate and associated *z* and *p* values, with classical visual significativity indication. Data for English has been previously reported in^19^.

Row	Comparison	French			English		
		Est.	*z*	*p*	Est.	*z*	*p*
1	ISO.acc, NAT	$-0.509$	$-10.94$	$<0.001$***	$-0.545$	$-12.16$	$<0.001$***
2	ANI.acc, NAT	$-0.420$	$-9.09$	$<0.001$***	$-0.722$	$-16.06$	$<0.001$***
3	ISO.syl, NAT	$-0.862$	$-18.45$	$<0.001$***	$-1.017$	$-22.40$	$<0.001$***
4	ANI.syl, NAT	$-0.820$	$-17.59$	$<0.001$***	$-1.127$	$-24.68$	$<0.001$***
5	ISO.acc, ANI.acc	$-0.088$	$-1.93$	0.273	0.177	4.00	$<0.001$***
6	ISO.syl, ANI.syl	$-0.042$	$-0.91$	0.878	0.110	2.43	$0.092^{.}$
7	ISO, ANI	$-0.130$	$-2.01$	0.233	0.287	4.54	$<0.001$***
8	syl, acc	$-0.753$	$-11.59$	$<0.001$***	$-0.878$	$-13.80$	$<0.001$***

As is verified in Fig. 1 and in the first 4 rows of Table 1, intelligibility of unmodified naturally timed sentences for French was significantly higher than sentences in any temporally-modified conditions. This result replicates what was obtained for English, and confirms that any temporal distortion leads to degraded intelligibility. In contrast to English however, where accent-isochronously retimed sentences were significantly more intelligible than accent-anisochronously retimed ones, no such effect is observed for French (Table 1 row 5). Similarly, the tendency for an isochronous versus anisochronous intelligiblity difference at the syllable level observed in English is absent in French (Table 1 row 6). Indeed, an overall benefit of isochronous over anisochronous transformation is observed for English but not in French, when combining the two rhythmic levels (Table 1 row 7).

As shown by the last row of Table 1, syllable level distortion led to greater intelligibility decrease, than accent-level distortion, in both French and English. This relates to the greater amount of distortion applied to syllable-level over the accent-level modifications applied to sentences, see Fig. 1, bottom panel.

In sum, while temporal distortion appears to be the main predictor of intelligibility for both languages, the independent role of isochrony seems to differ between both languages. We present in the next section evidence for a common pattern underlying these surface differences.

### Syllable-level isochrony plays a secondary role in both languages, even in naturally timed sentences

We defined several rhythm metrics to quantify, for each sentence in any temporal condition, the departure of either accent group or syllable timing from two canonical rhythm types: natural or isochronous rhythm. For the two hierarchical rhythmic levels considered here, this amounts to 4 metrics altogether: departure from naturally timed accent groups or syllables (respectively *dnat.acc* and *dnat.syl*), and departure from isochronous accent group or syllables (respectively *diso.acc* and *diso.syl*, see “Methods” and Table 5).

**Figure 2:**
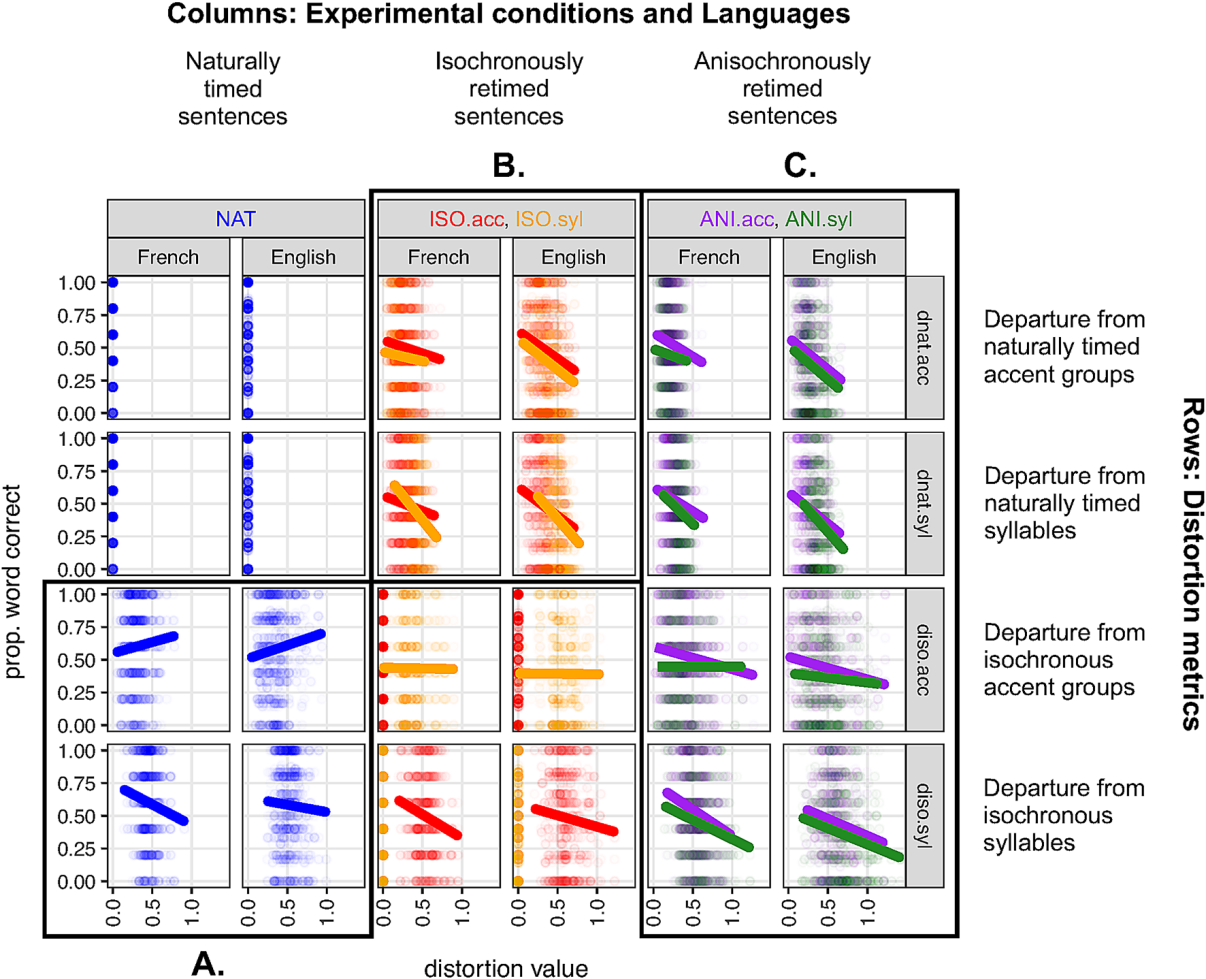
Intelligibility as a function of temporal distortion, as measured by the four metrics (rows) defined in Table 5. Data are grouped according to experimental condition (colors), the type of modification of the experimental condition (column groupings) and language (columns). Three subsets of data are highlighted for subsequent analysis (see text): (**A**) departure from isochrony of naturally timed sentences; (**B**) departure from natural rhythm of isochronously retimed sentences; (**C**) departure from natural rhythm and isochrony of anisochronously retimed sentences. Regression lines show linear modelling of the data points, disregarding subject and sentence random variation.

Figure 2 shows intelligibility scores as a function of the temporal distortion applied to the sentences along the 4 metrics, for all 5 experimental conditions, for English and French.

We analysed the joint role of isochrony and naturality in the different temporal conditions using logistic regression modelling (see “Methods”). We conducted three separate analyses by grouping natural, isochronous and anisochronous sentences respectively. This was done to avoid including subsets of data where a given metric would yield a zero-value by design (see Fig. 2). The three analyses are presented in the next subsections, each corresponding to a highlighted region in Fig. 2.

### Departure from isochrony in naturally timed sentences (Fig. 2 region A)

Naturally-timed sentences have a null departure from naturality by design, but their departure from an isochronous form at both the accent and the syllable level can be evaluated by the *diso.acc* and *diso.syl* metrics respectively. We therefore included in the analysis the *diso.acc* and *diso.syl* metrics, and discarded the *dnat.acc* and *dnat.syl* metrics (see Fig. 2 region A). Starting from the initial logistic regression model predicting intellibility with the full interaction of *language* (French and English), *diso.acc* and *diso.syl*, as fixed effects, we found that the simplest equivalent model was a model with only *diso.acc* and *diso.syl* factors without interaction (see Table 2 and “Methods”).

**Table 2:** (**A**) Initial (m1) and equivalent simpler (m2) model for the role of departure from isochrony in naturally timed sentences. The formulae of the fixed effects are given for the two models, and the result of a likelihood-ratio test between the two models is given on the right of the vertical separator. (**B**) m2 model coefficients, with associated *p* values. (**C**) Fixed-effect sizes with lower and upper confidence levels.

	Model summary		Likelihood-ratio test (m1, m2)		
	Fixed effects	AIC	$\chi ^2$	Df	$p(>\chi ^2)$
**(A) Model selection**					
m1	*language* $*$ *diso.acc* $*$ *diso.syl*	6728.4	3.36	5	0.65
m2	*diso.acc*+ *diso.syl*	6721.7			

	Estimate	SE	z value	Pr(>|z|)	
**(B) Equivalent model (m2) coefficients**					
(Intercept)	0.8633	0.3230	2.673	0.00752**	
*diso.acc*	1.0651	0.4633	2.299	0.02150*	
*diso.syl*	$-1.5151$	0.5757	$-2.632$	0.00850**	

Effect	$R^2$	Lower CL	Upper CL		
**(C) Fixed-effects size**					
m2	0.028	0.014	0.048		
*diso.syl*	0.018	0.006	0.034		
*diso.acc*	0.013	0.004	0.028		

The resulting model shows that for natural sentences, intelligibility is positively correlated with departure from accent isochrony (i.e., increased accent group irregularity is associated with better intelligibility) and negatively correlated with departure from syllable isochrony (i.e., the more isochronous naturally timed syllables are, the better the sentence is recognised). Importantly, this result does not depend on the language considered, with both French and English showing the same pattern of results. Fixed effect sizes are markedly small, as the majority of the variance is explained by random effects, as expected with the material used here. But fixed effects are nevertheless real and quantitatively important, as seen on Fig. 2.

### Departure from natural timing in isochronously retimed sentences (Fig. 2 region B)

Next we assessed to what extent intelligibility in isochronous conditions can be predicted from the departure from natural rhythm, at the accent and syllable levels (see Fig. 2 region B). From an initial fully interactional model with *language*, *dnat.acc* and *dnat.syl* predictors, the simplest equivalent model consisted of only the *dnat.syl* factor (see Table 3).

**Table 3:** (**A**) Initial (m3) and equivalent simpler (m4) models for the role of departure from natural rhythm in isochronously retimed sentences.The formulae of the fixed effects are given for the two models, and the result of a likelihood-ratio test between the two models is given on the right of the vertical separator. (**B**) m4 model coefficients, with associated *p* values. (**C**) Fixed-effect sizes with lower and upper confidence levels.

	Model summary		Likelihood-ratio test (m3, m4)		
	Fixed effects	AIC	$\chi ^2$	Df	$p(>\chi ^2)$
**(A) Model selection**					
m3	*language* $*$ *dnat.acc* $*$ *dnat.syl*	13,507	6.12	6	0.41
m4	*dnat.syl*	13,502			

	Estimate	SE	z value	Pr(>|z|)	
**(B) Equivalent model (m4) coefficients**					
(Intercept)	0.7382	0.1424	5.185	$2.16\mathrm{e}{-}07$***	
*dnat.syl*	$-2.6231$	0.2749	$-9.541$	$< 2\mathrm{e}{-}16$***	

Effect	$R^2$	Lower CL	Upper CL		
**(C) Fixed-effects size**					
m4	0.045	0.033	0.058		
*dnat.syl*	0.045	0.033	0.058		

This indicates that in conditions where sentences are isochronously transformed, intelligibility is significantly negatively correlated with departure from natural syllabic rhythmicity. Crucially, departure from accent group natural rhythm does not play a role, and the results are identical for both languages considered.

### Departure from isochrony and natural timing in anisochronously retimed sentences (Fig. 2 region C)

In this last step we evaluated whether intelligibility of anisochronously retimed sentences could be predicted by a combination of the four rhythmic distortion metrics (*diso.acc*, *diso.syl*, *dnat.acc* and *dnat.syl*, see Fig. 2 region C). Indeed, anisochronously retimed speech departs from both natural and isochronous canonical forms of timing, and in particular all four metrics have non all-zero values for these sentences. From an initial fully interactional model crossing *language* with the four rhythm metrics, we found that the simplest equivalent model consisted of the additive model of factors *dnat.syl* and *diso.syl* (see Table 4).

**Table 4:** (**A**) Initial (m5) and equivalent simpler (m6) model for the role of departure from isochrony and natural rhythm in anisochronously retimed sentences. The formulae of the fixed effects are given for the two models, and the result of a likelihood-ratio test between the two models is given on the right of the vertical separator. (**B**) m6 model coefficients, with associated *p* values. (**C**) Fixed-effect sizes with lower and upper confidence levels.

	Model summary		Likelihood-ratio test (m5, m6)		
	Fixed effects	AIC	$\chi ^2$	Df	$p(>\chi ^2)$
**(A) Model selection**					
m5	*language* $*$ *dnat.acc* $*$ *dnat.syl* $*$ *diso.acc* $*$ *diso.syl*	13,610	39.35	29	0.095
m6	*dnat.syl*+ *diso.syl*	13,591			

	Estimate	SE	z value	Pr(>|z|)	
**(B) Equivalent model (m6) coefficients**					
(Intercept)	0.9614	0.1669	5.760	$8.38\mathrm{e}{-}09$***	
*dnat.syl*	$-2.9418$	0.3852	$-7.637$	$2.22\mathrm{e}{-}14$***	
*diso.syl*	$-0.5311$	0.2742	$-1.937$	$0.0527^{.}$	

Effect	$R^2$	Lower CL	Upper CL		
**(C) Fixed-effects size**					
m6	0.059	0.046	0.073		
*dnat.syl*	0.030	0.021	0.041		
*diso.syl*	0.003	0.000	0.007		

These results refine and extend what was obtained in the previous two analyses. First, the rhythmic unit of accent group (whether defined at the stressed syllable level in English or at the accentual phrase level in French) does not provide any explanatory power in predicting intelligibility in anisochronously timed speech. Second, the role of natural syllable timing is confirmed and is the strongest predictor of intelligibility in that model as shown by its z-value (Table 4B) and its effect size (Table 4C). Third, a role for the departure from isochronously-timed syllables is detected. This means that in these conditions where the timing of speech is most unpredictable, there is a tendency for isochronous syllables to be associated with increased intelligibility. We note however that there is a necessary correlation between *dnat* and *diso* metrics for anisochronous sentences, exemplified by the fact that a close-to-isochronous natural sentence has to be distorted by a low *diso* value to be rendered isochronous, and that its anisochronous counterpart, being distorted by the same quantity by design, will be close to both the natural and the isochronous version. A quantitative analysis confirmed this (Pearson’s product-moment correlation between *diso.syl* and *dnat.syl*: French: $r=0.72, p<0.01$; English: $r=0.56, p<0.01$). The specific contribution of syllabic isochrony to intelligibility therefore appears to be small at best for anisochronous sentences. Finally, as for above analyses, this pattern of result applies indistinctively to both French and English.

## Discussion

In the current study, we set to characterise the possible role of isochrony in speech perception in noise. Isochrony is contrasted to naturalness, where the former refers to an ideally perfectly regular timing of speech units, while the latter to the timing of speech units as they occur in naturally produced speech. We included a third set of anisochronous conditions, in which the timing of speech events bear the same degree of temporal distortion from naturally timed speech as isochronous speech, while being irregular. We tested temporally modified sentences at the *accent* and the *syllable* levels, two hierarchically nested linguistic levels in English and French. These two languages are traditionally described as being representative of two distinct rhythmic classes, based on a hypothetical underlying isochrony of accent versus syllable units respectively in natural speech production^12,13,20^.

A first important result of this study is the replication from English to French that isochronous forms of speech are always less intelligible than naturally timed speech. In fact, in a paradigm as the current one, where the internal rhythmic structure of speech is changed but the sentence duration remains the same, any temporal distortion to the naturally produced timings of speech units appears to be detrimental, with the amount of temporal distortion being a strong predictor of intelligibility decrease. This result goes against the hypothesis that isochronous speech, by virtue of a supposedly ideally regular timing of its units, would be easier to track, by alleviating the need for constant phase-resetting. For traditional linguistic accounts too, these results further debunk the isochrony hypothesis, assuming that produced speech would be based on an underlying ideally isochronous form^12^, see review in^13,20^. In fact, our results suggest that the natural timing statistics are actively used by listeners in decoding words, even though they are encountered in a single instance by listeners.

At the condition level, while an advantage of isochronous over anisochronous forms of speech was found for English^19^, no such trend was observed for French. While possible prosodic or idiosyncratic effects might account for this difference (see Supplementary Materials), this led us to examine, separately by condition, the relationship between intelligibility and the four timing metrics, namely departure from the natural timing of speech units and departure from a hypothetical underlying isochronous form of those speech units. This analysis unveiled a rather consistent portrait across the two talkers of the two languages, according to the type of sentence retiming. For naturally-timed sentences, intelligibility correlates with the degree of departure from an ideal isochronous form of the sentence at the syllable level. That is, naturally isochronous sentences at the syllable level are significantly better recognised than naturally anisochronous sentences. For isochronously retimed sentences, intelligibility was strongly correlated with departure from naturality (see also Fig. 1, bottom). Importantly, this result is valid only for the syllable level—departure from natural timing of accent groups did not explain intelligibility variation. For anisochronous sentences, the two syllabic rhythm metrics, that is, departure from naturally timed syllabes and departure from syllabic isochrony, were found to be correlated with intelligibility, though the latter to a much lesser extent. This shows that both temporal dimensions of syllabic rhythm are actively relied upon in speech comprehension. In addition, the simultaneous variation of the two temporal dimensions for this type of stimuli enabled to provide an indication of the relative size of these effects, since the role of departure from naturally timed syllables was about ten times stronger than the role of departure from isochronously timed syllables (Table 4C)—a ratio however possibly underestimated owing to the necessary correlation that exists between the two metrics, by design for these sentences. Again, the regularity of accent group timing did not provide any significant increase in intelligibility in any condition—contrary to natural sentences, as we will discuss later.

Crucially, all three analyses did not find language as a contributing factor in explaining intelligibility, with the same pattern of result applying to speech produced by both English and French talkers. Of course, as we tested only one talker in each language, there is the possibility that an observed difference (or lack thereof) between languages might be imputable to confounded talker characteristics (see Supplementary Material for an analysis of talker idiosyncrasies on a range of acoustic parameters). With that caveat in mind, given that those languages were selected as being representative of different rhythmic classes, the undiscriminative nature of the results may have two implications. First, it suggests that isochrony effects could apply across the board to any language, independently of their rhythmic patterning at a global level—though, of course, this will have to be tested on other languages and multiple speakers in future studies. Second, our results suggest that the syllable is a core unit in temporal speech processing. While the primary role for the syllable in speech processing has long been proposed^7,21,22^, (but see^8^) the fact that isochrony effects are stronger for syllable than accent groups even for a stress-timed language (which would supposedly favour accent-group isochrony), indicates that the temporal scale associated with the syllable is key—rather than its linguistic functional value. Instead, our results further support the usefulness of the notion of a neurolinguistic unit defined at the crossroads of linguistic and neurophysiological constraints, such as the so-called “theta-syllable”^23^ which would apply universally to languages independently of their linguistically-defined rhythm class. We suggest that this unit combines high-level perceptually-grounded temporal anchoring, with the P-centre being a good candidate, with physiologically-based temporal constraints determined by neural processing in the theta range of 4–8 Hz (or up to 10 Hz, see^24,25^).

Taken together, the data presented here are compatible with a model of cortical organisation of speech processing with co-occurring and inter-dependent bottom-up and top-down activity^25,26^. While early studies have focussed on exogenous effect of rhythm on cortical activity, the role of endogenous activity is being progressively acknowledged and understood^27^. In this study, we use intelligibity as a proxy for successful cortical processing, following the long established link between intelligibility and entrainment^28–30^. Our data allow, across two languages with markedly different rhythmic structures, to suggest unifying principles: the dependence of intelligibility of naturally-timed sentences to their underlying isochrony suggests a bottom-up response component that benefits from regularity at the syllable level. In isochronous sentences, the strong response variation with departure from the sentences’ natural rhythm indicates that learned patterns are a strong determinant of speech processing. Unifying both distortion types, anisochronous sentences display a graded dependence to both of temporal distortions, and additionally provide a quantification of the relative magnitude of the effects, since in this type of sentences, top-down processing has a stronger role than bottom-up information. These hypotheses need to be backed-up with neurophysiological data, and a first work in that framework is reported in^31^.

A point of interest of our results is the positive correlation in natural sentences between intelligibility and departure from accent-level isochrony in both English and French. Local intelligibility variations associated with regular versus irregular accent-level prominences could explain this effect: irregular accent-level units in a sentence might trigger fewer but louder prominences in a sentence compared to isochronously distributed accent units, which in turn may prove more intelligibile when mixed with a fixed signal-to noise ratio over the entire sentence, as opposed to a more uniform distribution of energetic masking over the sentences. The psychoacoustic consequences of this hypothesis are difficult to control and outside the scope of the current study but could open productive avenues in speech intelligibility enhancing modifications^32^.

The sentence-long speech material employed here can be considered a good match to ecologically valid speech perception conditions (over more classical controlled tasks involving lexical decision or segment-level categorical perception^33^). In everyday communicative settings however, listeners do make use of a much larger temporal context for successfully decoding speech, efficiently incorporating semantic, prosodic or discursive cues from a temporal span in the range of minutes rather than seconds. It is however difficult to predict the potential effect that isochronously retimed speech might produce in a broader temporal context, as it may become an *explicit* feature of the stimulus to be perceived. Indeed, none of the participants in the current study reported the isochronous quality of modified speech, but with sustained stimulation beyond the duration of a sentence, listeners may be able to project a temporal canvas onto upcoming speech. One could hypothesise both a facilitatory effect due to the explicit nature of the isochronous timing, as found in rhythmic priming studies^34–36^, but also a detrimental effect due to the sustained departure from optimally-timed delivery of information found in natural speech, as defended here. The relevance of examining long-term oscillatory entrainment in the delta and theta range also needs to be posed, although phase-locking effects beyond the duration of a phrase seem unlikely^37,38^.

In conclusion, in light of the data presented here, we propose an account of the role of isochrony in speech perception which unifies previous hypotheses on the role of isochrony in speech production and perception^39,40^ but, importantly, quantifies its contribution, with respect to the “elephant-in-the-room” factor, that is, natural timing of speech. First, to avoid any ambiguity, we propose that isochrony does not have a primary role in speech perception or production. Indeed, through the effect of the temporal manipulations of speech reported here, it is clear that isochronous forms of speech do not provide a benefit in speech recognition in noise. In fact, the natural timing statistics of speech appear to contain an essential ingredient used by listeners in the form of learned information, to which listeners attend to by recruiting top-down cortical activity, and allows them to process sentences even though they are presented for the first time and degraded by noise. We however show that isochrony does play a role, albeit a secondary one, which is compatible with a by-product output of neuronal models of oscillatory activity^41–43^. Importantly, the theta-syllable provides the pivot for isochrony in speech and oscillations in the brain in this context. Entrainment to oscillatory activity has received a lot of attention in the recent years and received broad experimental support, in particular in specific experimental settings where the material is explicitely presented in an isochronous form (in visual, auditory, musical contexts^38,44–47^). The fact isochrony effects are observed in a context where isochrony is not an explicit characteristic of the experimental material suggests that these effects could reflect physiological mechanisms which, if sustained, can lead to the kind of behavioural outcomes reported in controlled setups. In sum, we suggest that isochrony is not a requirement for successful speech processing but rather a kind of underlying timing prior actively suppressed by the necessities of flexible and efficient linguistic information encoding. Because of plausible neuroanatomical architecture of cortical processing however, isochrony seems to represents a canonical form of timing that can be seen as an attractor, and thereby enjoys a special status in perception and production generally, such as poetry, music and dance.

## Methods

### Speech material and annotation

The speech material consisted of sentences, taken from the Harvard corpus for English^15^ and the Fharvard corpus for French^16^. Both corpora are highly comparable in their structure: they both contain 700 sentences or more, each sentence is composed of 5–7 keywords (exactly 5 keywords for French), with a mild overall semantic predictability, and sentences are phonetically balanced into lists of 10 sentences. For the current study, we used a subset of 180 sentences for each corpus, recorded by a female talker for English^48^ and by a male talker for French^49^. Each sentence subset was randomly sampled from all sentences of each corpus.

Sentences were annotated at two hierarchical rhythmic levels: the *accent group* and the *syllable* level. The syllable level was taken as the lowest hierarchical rhythmical unit in both languages, and the accent group was defined as the next hierarchical step up the rhythmic hierarchy, i.e., the stressed-syllable in English and the accentual phrase in French^50,51^. While annotation of stressed syllables in English was relatively straightforward, accentual phrase boundaries prove more difficult to determine unambiguously, as they combine several factors, from the syntactic structure of the sentence to the particular intonational pattern used by the talker producing the sentence. We relied on an independent annotation of accent group boundaries done by three native French speakers. From an initial set of 280 sentences, 181 sentences received a full inter-annotator agreement, and the first 180 sentences of that set were selected for the current study.

The P-centre associated to a rhythmic unit corresponds tothe onset of the vowel associated with that unit in the case of simple consonant-vowel syllables, and is usually advanced in the case of complex syllable onsets, or when the vowel is preceded by a semi-vowel. In both corpora, P-centre events were first automatically positioned following an automatic forced alignement procedure^52^, then manually checked and corrected if necessary, typically when a schwa was inserted or deleted in a given syllable. Given the hierarchical relationship between accent groups and syllables, the accent group P-centre aligns with the corresponding syllable P-centre. See Supplementary Materials for auditory examples of annotated sentences at the accent group and syllable levels.

### Stimuli

Sentences were temporally modified by locally accelerating or slowing down adjacent successive speech segments. Unmodified time onsets of either accent (acc) or syllable (syl) rhythmic units served as a reference for the natural rhythm (NAT) condition, from which isochronous (ISO) and anisochronous (ANI) conditions were defined, as detailed below.

We first defined *t* the *reference* time series identifying the time onsets of the *N* corresponding rhythmic units of a sentence flanked with the sentence endpoints, i.e., $t=t_0, t_1, \ldots , t_N, t_{N+1}$, with $t_0$ and $t_{N+1}$ respectively the start and end of the sentence. We noted *d* the associated inter-rhythmic unit durations, i.e., $d_i=t_{i+1}-t_{i}, i=0, \ldots , N$.

We then defined the *target* time series $t'$ and the associated durations $d'$, the resulting values after temporal transformation. The initial and final portions of the sentence were left unchanged, i.e., $t'_0=t_0, t'_1=t_1, t'_N=t_N$ and $t'_{N+1} = t_{N+1}$, hence $d'_0=d_0$, and $d'_{N}=d_{N}$. For the ISO condition, the target durations were set to the average duration of the corresponding intervals in the reference time series NAT, i.e., $d_{i}'=\frac{t_N-t_1}{N-1}$, $i=1, \ldots , N-1$. For the ANI condition, we imposed that sentences were temporally transformed by the same quantity as isochronous sentences, but resulted in an unpredictable rhythm. We achieved this by using the following simple heuristic, consisting of applying an isochronous transformation to the *time-reversed* rhythmic units events. First, the reference time series was replaced by a pseudo reference time series made of pseudo events such that successive pseudo reference durations were the time reversal of the original reference durations, i.e., $\text {rev}(d_{i})=d_{{N-i}}, i=1, \ldots , N-1$; then, the target time series ANI were computed from this reversed sequence by equalising the temporal distance of the pseudo events as in the ISO condition.

Temporal transformation was then operated by linearly compressing or expanding successive speech segments identified by the reference time series by applying the duration ratio of target to reference speech segments, i.e., applying a time-scale step function $\tau _i=\frac{d'_i}{d_i}, i=1, \ldots , N$ to the speech signal of the sentence. This was achieved using WSOLA^53^, a high-quality pitch-preserving temporal transformation algorithm.

**Figure 3:**
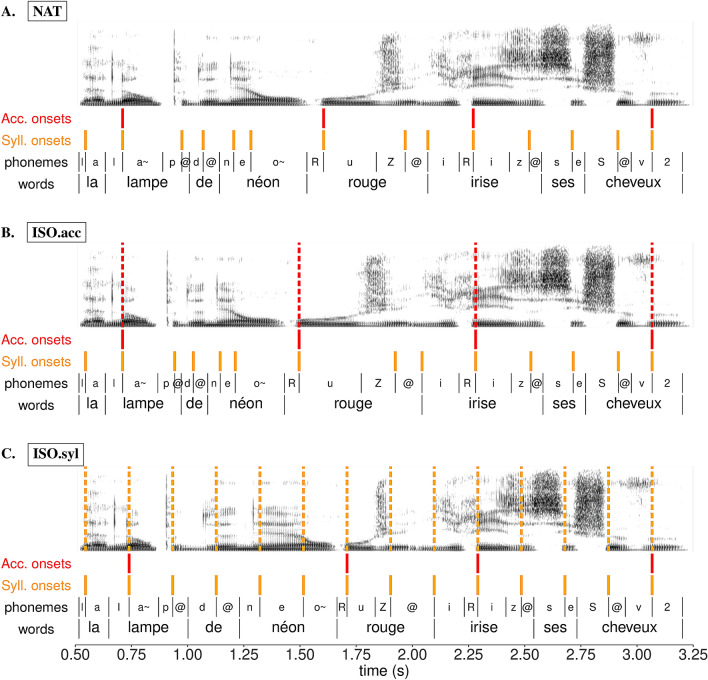
Annotation of an example sentence (translation: *The red neon lamp makes his/her hair iridescent*), in its original unmodified natural timing (**A**) and transformed isochronous forms at the accent **(B)** and syllable **(C)** levels. For each panel, from top to bottom: spectrogram with target boundaries used for the transformation overlaid in dashed lines, accent group onsets (red), syllable onsets (orange), phonemes and words.

Altogether, we obtained 5 temporal versions of each sentence in the corpus: the unmodified natural version (**NAT**), the isochronous stimuli at the accent (**ISO.acc**) and syllable (**ISO.syl**) levels, and the anisochronous stimuli at the accent (**ANI.acc**) and syllable (**ANI.syl**) levels. Figure 3 shows the result of the temporal transformation of an example sentence for the first 3 conditions. See Supplementary Materials for examples of retimed stimuli.

Final experimental stimuli were constructed by mixing the temporally transformed sentence with speech-shaped noise at a signal-to-noise ratio of $-3$ dB. This value was determined in previous studies^16^ to elicit a keyword recognition score of around 60% in the unmodified speech condition, in turn providing maximum sensitivity for the difference between unmodified and modified speech conditions. Speech-shaped noise was constructed, separately for each talker, by filtering white noise with a 200-pole LPC filter computed on the concatenation of all sentences of the corpus recorded by that talker.

### Temporal distortion metrics

We quantified the net amount of temporal distortion $\delta$ applied to a given sentence by computing the root mean square of the log-transformed time-scale step function $\tau$ associated to that sentence. Log-transformation was done so that compression and elongation by inverse values (e.g., $\times \frac{1}{2}$ and $\times 2$ respectively) would contribute equally to the overall distortion measure (i.e., ${log(\frac{1}{2})}^2={log(2)}^2$). Binary logarithm was used here. Using our notation referring to discrete events, the temporal distortion from the reference time series *t* with *N* events to the target time series $t'$ can therefore be written:

1 $$\begin{aligned} \delta = \sqrt{\frac{\sum _{i=1}^{N}{(\log {\tau _i})^2} d_i}{\sum _{i=1}^{N}{d_i}}} \end{aligned}$$

where $i=1,\ldots ,N$ is the event index, $\tau$ the time-scale factors linking *t* and $t'$ and *d* the duration between successive reference events, as defined in the preceding section. Note that the $d_i$ term in the numerator emerges from the grouping of samples between successive reference events, since for all these samples $\tau _i$ values are constant by design.

By design, individual sentences undergo an identical amount of temporal distortion in isochronous and anisochronous transformations. By extending the application of the $\delta$ function to time instants other than the ones used for stimulus construction, we introduce additional metrics to evaluate the departure of the rhythm of any sentence – whether temporally modified or not – to two canonical rhythm types associated with the sentence: its unmodified natural rhythm and its isochronous counterpart. For the two hierarchical rhythmic levels considered here, this amounts to 4 new metrics: departure from naturally timed accent groups or syllables (respectively *dnat.acc* and *dnat.syl*), and departure from isochronous accent group or syllables (respectively *diso.acc* and *diso.syl*, see Table 5).

**Table 5:** Analysis metrics to evaluate departure from naturally-timed and isochronous forms at the accent and syllable level (rows) in each of the 5 experimental conditions (columns). Each argument of the $\delta$ function (Eq. 1) is a time series of either accent (acc) or syllable (syl) onsets, as they occur in a given experimental condition. For example, $t_{\text {syl}_{ISO.acc}}$ represents the syllable onsets of a sentence as they occur in the transformed ISO.acc experimental condition. Note that some of these distortions are equal to 0 by construction: they are *dnat.acc* and and *dnat.syl* for NAT sentences, and *diso.acc* and *diso.syl* for ISO.acc and ISO.syl sentences respectively.

	NAT	ISO.acc	ISO.syl
*dnat.acc*	$\delta (t_{\text {acc}_{NAT}},\, t_{\text {acc}_{NAT}})$	$\delta (t_{\text {acc}_{NAT}},\, t_{\text {acc}_{ISO.acc}})$	$\delta (t_{\text {acc}_{NAT}},\, t_{\text {acc}_{ISO.syl}})$
*dnat.syl*	$\delta (t_{\text {syl}_{NAT}},\, t_{\text {syl}_{NAT}})$	$\delta (t_{\text {syl}_{NAT}},\, t_{\text {syl}_{ISO.acc}})$	$\delta (t_{\text {syl}_{NAT}},\, t_{\text {syl}_{ISO.syl}})$
*diso.acc*	$\delta (t_{\text {acc}_{ISO.acc}},\, t_{\text {acc}_{NAT}})$	$\delta (t_{\text {acc}_{ISO.acc}},\, t_{\text {acc}_{ISO.acc}})$	$\delta (t_{\text {acc}_{ISO.acc}},\, t_{\text {acc}_{ISO.syl}})$
*diso.syl*	$\delta (t_{\text {syl}_{ISO.syl}},\, t_{\text {syl}_{NAT}})$	$\delta (t_{\text {syl}_{ISO.syl}},\, t_{\text {syl}_{ISO.acc}})$	$\delta (t_{\text {syl}_{ISO.syl}},\, t_{\text {syl}_{ISO.syl}})$

		ANI.acc	ANI.syl
*dnat.acc*		$\delta (t_{\text {acc}_{NAT}},\, t_{\text {acc}_{ANI.acc}})$	$\delta (t_{\text {acc}_{NAT}},\, t_{\text {acc}_{ANI.syl}})$
*dnat.syl*		$\delta (t_{\text {syl}_{NAT}},\, t_{\text {syl}_{ANI.acc}})$	$\delta (t_{\text {syl}_{NAT}},\, t_{\text {syl}_{ANI.syl}})$
*diso.acc*		$\delta (t_{\text {acc}_{ISO.acc}},\, t_{\text {acc}_{ANI.acc}})$	$\delta (t_{\text {acc}_{ISO.acc}},\, t_{\text {acc}_{ANI.syl}})$
*diso.syl*		$\delta (t_{\text {syl}_{ISO.syl}},\, t_{\text {syl}_{ANI.acc}})$	$\delta (t_{\text {syl}_{ISO.syl}},\, t_{\text {syl}_{ANI.syl}})$

### Participants and procedure

Data for English are based on 26 participants (21 females) with mean age of 20.9 (SD = 6.3), all speaking Australian English as a native language, with no known audition troubles and are described in detail in^19^. We include the data for English previously reported in^19^ for the purpose of comparing with the French data. New analyses are conducted on this dataset (see “Results”). All experimental protocols were approved by the Human Research Ethics Committee of Western Sydney University under the reference H9495. For French, 27 participants (15 females), with mean age of 26.7 years (SD = 8.8) were recruited from the student and staff population of the University of Grenoble-Alpes (UGA) and were given a 15 euro gift card as compensation for their participation. We checked that all participants met the selection criteria which included speaking French as a native language, and having no known audition troubles. No data was removed from the initial set. All experimental protocols were approved by UGA’s ethical committee (CERGA, agreement IRB00010290-2017-12-12-33). For both English and French studies, all methods were carried out in accordance with relevant guidelines and regulations, and informed consent was obtained from all participants.

For both language groups, participants were given written instructions and the experimenter gave complementary information when necessary. Participants then sat in front of a computer screen, where short on-screen instructions were given before each block of the experiment. Participants were presented with speech and noise mixtures played binaurally through Beyer Dynamic DT 770 Pro 80 ohm closed headphones at a comfortable level set at the beginning of the experiment and maintained constant throughout the experiment. Participants had to type what they heard on a keyboard and press “Enter” to trigger the presentation of the next stimuli. Stimuli were grouped by condition forming 5 blocks of 36 sentences each. An additional 5 stimuli from each condition were presented as practice in the beginning of the experiment and were not used for further analysis. The order of conditions was counterbalanced across participants, and the order of sentences was pseudo-randomised for each participant. The order of the practice sentences was fixed across all participants, and the order of conditions for practice sentences was also fixed, to: NAT, ISO.acc, ANI.acc, ISO.syl, ANI.syl.

### Scoring

Sentences were automatically scored with custom-made scripts that matched keywords with listeners typed responses. A dictionary of variants was used in both languages to correct for homophones, full letter spelling of digits and common spelling mistakes (e.g., *‘ciggar’ corrected to ‘cigar’ in English, and ‘*cigne’ corrected to ‘cygne’ (‘*swan*’) in French. Each sentence received a final score as the proportion of keywords correctly recognised.

The accuracy of the automatic scoring was evaluated on a 530-sentence subset of the listeners responses (around 5.5% of all 9450 responses), randomly and uniformly sampled across subjects, conditions and languages. The subset was manually scored, and we found that 98% of the sentences were correctly scored by the automatic procedure, with the 10 incorrectly scored sentences each having no more than one word typed with a spelling mistake which was absent in the dictionary (and therefore added to it for future studies).

### Data modelling

The effect of experimental condition on intelligibility was analysed for French following previously reported analysis of the English data^19^. A generalised linear mixed-effect model (function glmer from the R package lme4^54^) was fitted to intelligibility scores, including a random term intercept by subject. Normal distribution of residuals was visually verified. Generalised simultaneous hypothesis were formulated and tested with the function glht from the R package multcomp^55^, which corrects for multiple comparisons.

Analysis of the contribution of the temporal distortion metrics to intelligibility was performed on combined French and English data. Three models were fitted for each of the three subsets of the data (see Fig. 2 and “Results”). Fixed effects were language (French and English) and the non all-zero valued metrics for the given data subset. Random effect structure included a term by sentence and by participant. For each model, we report an analysis of variance between the initial full model and the minimal equivalent model. The latter is obtained from the initial model by incrementally removing fixed-effect terms until no term can be removed without signicantly changing the explained variance. Visual distribution of residuals was checked. Fixed-effects size are computed with the function r2beta from the R packager2glmm^56^).

## Supplementary information

Supplementary Information 1.

Supplementary Information 2.

Supplementary Information 3.

## Data Availability

Example stimuli illustrating P-centre annotation and temporal modifiction as well as computer code for the temporal distortion metrics are included in *Supplementary Materials*. Experimental stimuli and listeners’ responses data are available for download at: https://doi.org/10.5281/zenodo.3966475.
