# Supplementary material for: The role of isochrony in speech perception in noise
Source: Sci Rep. 2020 Nov 11;10:19580. doi: 10.1038/s41598-020-76594-1 (PMC7658253; doi:10.1038/s41598-020-76594-1)
Supplement: Supplementary file 3 — Supplementary Information 3. [file 41598_2020_76594_MOESM3_ESM.pdf]

The role of isochrony in speech perception in noise

Vincent Aubanel[1,*] and Jean-Luc Schwartz[1]

[1]CNRS, GIPSA-lab, University of Grenoble Alpes, Grenoble, France
[*]vincent.aubanel@gipsa-lab.fr

Supplementary Materials

Example stimuli

The following audio files are available as Supplementary material. Note that these stimuli are example sentences to illustrate the P-centre annotation and temporal transformation. None of these files were heard as such by participants, who instead heard speech-plus-noise mixtures. Experimental stimuli and listener data is available at `https://doi.org/10.5281/zenodo.3966475`.

Unmodified example sentences

- `fr.wav` is an example sentence in French ("L'offre est limitée aux mille premiers clients" / *"The offer is limited to the first 1000 customers"*)
- `en.wav` is an example sentence in English ("The navy attacked the big task force")

Example stimuli illustrating P-centre annotation

- `fr_p_acc.wav` is the French example sentence mixed with audible tones marking accent-group P-centres
- `fr_p_syl.wav` is the French example sentence mixed with audible tones marking syllable P-centres
- `en_p_acc.wav` is the English example sentence mixed with audible tones marking accent-group P-centres
- `en_p_syl.wav` is the English example sentence mixed with audible tones marking syllable P-centres

Example stimuli illustrating temporal modifications

- `fr_iso_acc.wav` is the French example sentence isochronously retimed at the accent-group level
- `fr_iso_syl.wav` is the French example sentence isochronously retimed at the syllable level
- `fr_ani_acc.wav` is the French example sentence anisochronously retimed at the accent-group level
- `fr_ani_syl.wav` is the French example sentence anisochronously retimed at the syllable level
- `en_iso_acc.wav` is the English example sentence isochronously retimed at the accent-group level
- `en_iso_syl.wav` is the English example sentence isochronously retimed at the syllable level
- `en_ani_acc.wav` is the English example sentence anisochronously retimed at the accent-group level
- `en_ani_syl.wav` is the English example sentence anisochronously retimed at the syllable level

Word-level and talker acoustics analysis

Word-level intelligibility (Figure 1) and individual talker acoustic analysis (Figure 2) indicate that both syntactic differences in how the sentences are formed in the two langages, and individual talker's differences in speaking rate variation across sentences could be a contributing factor for a contrastive tendency across French and English.

As seen in Figure 1, the two languages present three marked differences. First, for NAT sentences, intelligibility drops sharply after the first word followed by an increase in French while it is highest for the first 3 words followed by a large decrease towards the end of the sentence in English. This pattern seems to hold in each language for other conditions as well, combined with an additional decrease in intelligibility. Second, the isochronous advantage established for English at the accent level appears to be developing in the second part of the sentence, that is, accent-isochronous sentences show a milder decrease as the sentences unfold compared to other conditions. Third, intelligibility of initial keywords in accent-retimed French sentences have similar levels than NAT speech. This could be attributed to the fact that because accent group boundaries tend to fall on the last syllable of words in French (contrasting with trochaic stress patterns in English), the early part of the retimed sentence is in effect unmodified until that point in these conditions. Taken together, these differences in word-level recognition patterns suggest that the syntactic and rhythmic structure of both languages are likely to have an effect on how isochronous modification are implemented by talkers and processed by listeners.

As seen in Figure 2, there is a wider F_0 declination and a stronger intensity declination in English as opposed to French, potentially due to gender difference and individual speaking style. There is also a sensible difference across French and

English in average speaking rate in both accent group and syllable levels. Finally, we note that speaking rate is more irregular across the sentence for the female talker, especially at the syllable level. In sum, both idiosyncratic and language factors seem to underly differences in the three acoustic descriptors. These prosodic variations in English, with faster rate and lesser intensity towards the end of the sentences, could well explain the effect of keyword position in Figure 1.

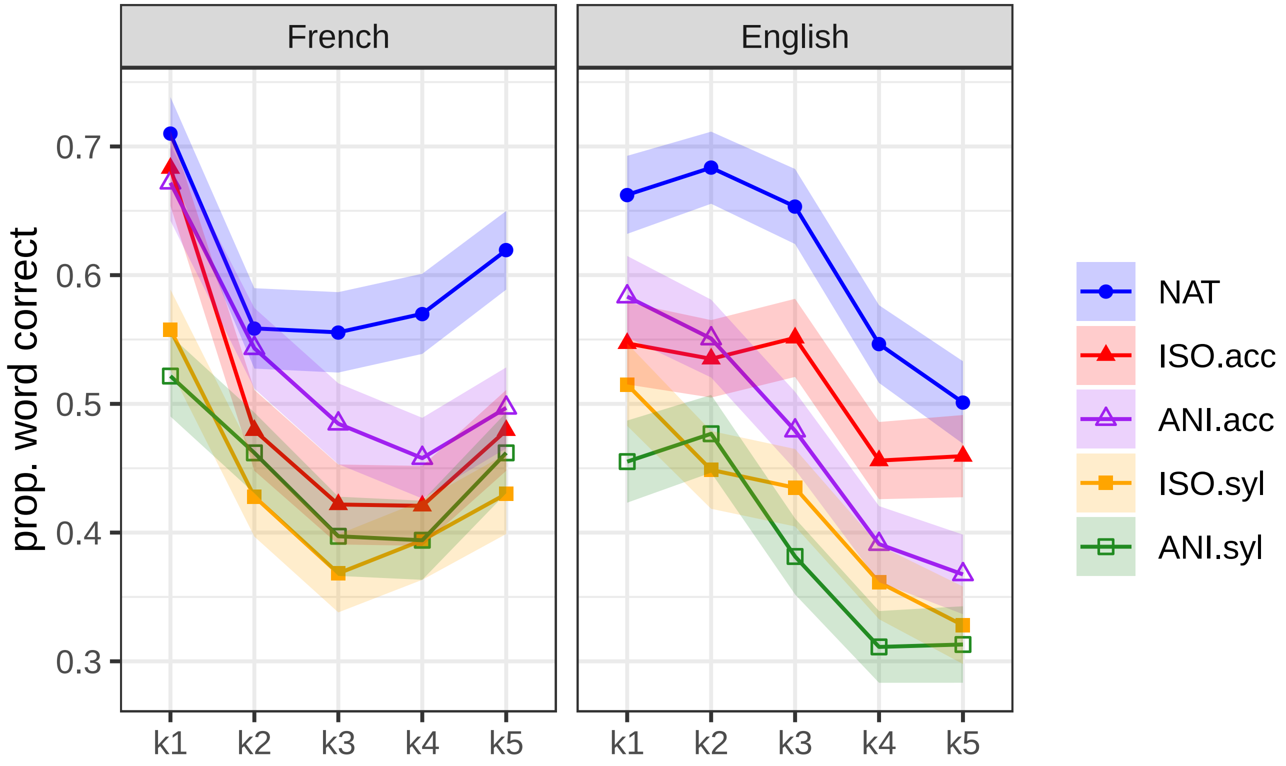

Figure 1. Intelligibility scores split by keyword, for French and English. Ribbons indicate 95% confidence interval over subjects.

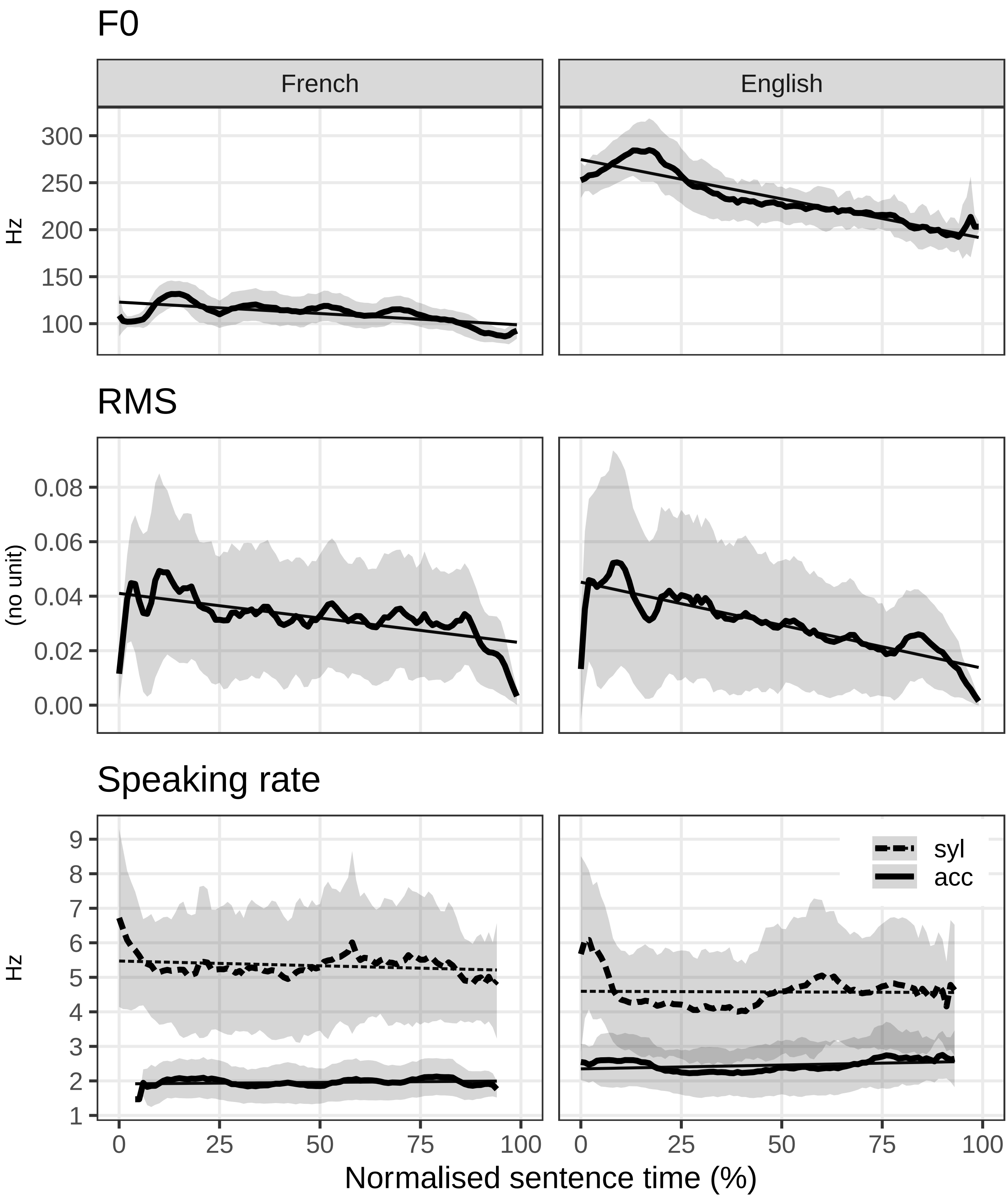

Figure 2. Comparison of fundamental frequency (F_0), root-mean square energy and speaking rate for the male French talker and the female English talker used in the study. Ribbons indicate 95% confidence interval over sentences (N=180 in each language).
